# 

**Published:** 1855-03

**Authors:** 


*Ranking's Abstract*, for January, is received from Messrs. Lindsay and Blakiston, Philadelphia.

It contains the usual amount of valuable matter, selected from all quarters. Among the American extracts we notice medical papers by Drs. Storer, of Boston, Purple and Swift, of New York, and surgical papers by Drs. Mott and Markoe, of New York, and the article on "Elkoplasty," of our colleague Prof. Hamilton, of Buffalo.

The number seems to be full of interest, and is a very valuable though cheap addition to the library of the physician. H.

---

The next number will contain a paper on Iodide of Iron, by Dr. Nelson, the inaugural thesis of Wm. Howell, and sundry notices of books just received. H.

---

We do not exactly "stop the press to announce," but we use the fag end of the last page, to inform our readers that a libel suit has been commenced by Mr. J. D. Hill against the editors of this Journal, for an article signed 'Justice," and published in the No. of last October. H.

